# Shared decision making and associated factors among patients with psychotic disorders: a cross-sectional study

**DOI:** 10.1186/s12888-023-05257-y

**Published:** 2023-10-13

**Authors:** Espen W. Haugom, Jūratė Šaltytė Benth, Bjørn Stensrud, Torleif Ruud, Thomas Clausen, Anne Signe Landheim

**Affiliations:** 1 Norwegian National Advisory Unit on Concurrent Substance Abuse and Mental Health Disorders, Innlandet Hospital Trust, P.B 104, Brumunddal, 2381 Norway; 2 Norwegian Centre for Addiction Research (SERAF), Institute of Clinical Medicine, University of Oslo, Blindern, Oslo Norway; 3 Division of Mental Health Services, Akershus University Hospital, Lørenskog, Norway; 4 Institute of Clinical Medicine, University of Oslo, Oslo, Norway; 5 Health Services Research Unit, Akershus University Hospital, P.O. Box 1000, 1478 Lørenskog, Norway

**Keywords:** Shared decision making, Psychotic disorders, CollaboRATE, Mental health care

## Abstract

**Background:**

Shared decision making is a process where patients and clinicians collaborate to make treatment choices based on the patients’ preferences and best available evidence. The implementation of shared decision making remains limited for patients with psychotic disorders despite being recommended at policy level, being advocated as ethical right and wanted by the patient’s. A barrier to implementation that is often mentioned is reduced decision-making capacity among patients. The challenges of implementing shared decision making highlights a need for more knowledge on shared decision making for these patients. Moreover, the association between patient-related characteristics and shared decision making is unclear, and further research have been suggested. More knowledge of factors associated with involvement in shared decision making can enhance understanding and help to empower patients in the decision-making process. The current study examined the extent of reported shared decision making among patients with a psychotic disorder in mental health care and factors associated with shared decision making.

**Methods:**

This study included 305 participants with a psychotic disorder from 39 clinical inpatient and outpatient sites across Norway. Shared decision making was assessed using the CollaboRATE survey. A linear mixed model was estimated to assess characteristics associated with shared decision making scores.

**Results:**

The CollaboRATE mean score was 6.3 (ranging from 0 to 9), the top score was 14.1% and increased global satisfaction with services was significantly associated with a higher level of shared decision making (regression coefficient 0.27, 95% confidence interval (0.23; 0.32), p < 0.001).

**Conclusions:**

The low top score shows that few patients felt that they received the highest possible quality of shared decision making, indicating that many patients found room for improvement. This suggests that services for patients with psychotic disorders should be designed to give them a greater role in decision making. Shared decision making might play a key role in mental health care, ensuring that patients with psychotic disorders are satisfied with the services provided.

**Trial registration:**

NCT03271242, date of registration: 5 Sept. 2017.

## Background

Decision-making models can be described on a continuum from paternalistic to informed, with shared decision making (SDM) as an intermediate model [1]. SDM is an approach where as a minimum the patient and clinician collaborate to make decisions based on the patient’s preferences and clinical evidence [2, 3]. There are two kinds of experts in the SDM process. Patients are experts on their own life and provide information on what is important for them. Health professionals have expert knowledge on evidence-based treatment options and should inform patients about the advantages and disadvantages of the different choices [3]. In SDM, these two types of expert work together, sharing information, discussing alternatives and comparing options, aiming to arrive at a preference-based decision that respects what matters most to the patient [4]. This process may also include other health and social care professionals and people from the social network of the patient such as family members or peer support workers [5].

Several conceptual definitions have been suggested since the SDM model was introduced in 1982 [6]. Charles and colleagues made an early and widely cited contribution [7]. They described the following four key characteristics of SDM; *that at least two participants - physician and patient - be involved, that both parties share information, that both parties take steps to build a consensus about the preferred treatment, and that an agreement is reached on the treatment to implement* [7]. The integrative model of SDM by Makoul and Clayman [8] showed that patients’ values, preferences, and options are the most common elements used in SDM definitions. Meanwhile, the more recent “three-talk model of SDM” proposed by Elwyn and colleagues [4] includes the three steps of “team talk”, “option talk”, and “decision talk”. In team talk, choices are described, support are offered and goals are explored. Options talk involves discussing and comparing potential choices, and decision talk is the step where a preference-based decision is made based on what matters most to the patient.

Conceptual frameworks and definitions of SDM in mental health care have also been suggested. These perspectives see SDM not just as a one-time consultation, but also as a practice that may potentially involve a variety of related processes [9, 10]. Consequently, SDM can be viewed as an ongoing process, where elements like continuity of care and building a relationship based on trust and honesty are important [11].

The form of collaboration that SDM involves may improve patient satisfaction and decrease decisional conflict [12]. Research has also shown that SDM can promote greater patient engagement, increase knowledge about services, and help to enhance recovery and hope in individuals with serious mental illness [13]. This can be seen in line with the aim of SDM, which is to decrease traditional power asymmetry and strengthen patient information, autonomy and patients’ decisional position [7, 14].

Implementation of SDM in mental health care remains limited, even though the model is recommended at the policy level, advocated as the ethically right thing to do [1] and desired by people with a psychotic disorder [15, 16]. A review shows that SDM is not routinely implemented for people with severe mental illnesses [17] and previous studies have also found that patients with psychotic disorders report a lower extent of SDM than do patients with other mental health diagnoses [18, 19]. The fact that patients with psychotic disorders participate less in care decisions than they desire underlines a need for more knowledge of SDM for these patients.

Several barriers may hinder implementation. A review on trends and perspectives of SDM in schizophrenia and related disorders found that health professionals consider patients’ reduced decisional capacity and time constraints as barriers [20]. This is supported by another review focusing on people with severe mental illness, where the authors also found that a diagnosis of schizophrenia and the presence of severe symptoms were barriers, as SDM was less likely to occur in such circumstances [17]. A qualitative study of the views of clinicians and service users has shown that a barrier to SDM in psychiatric medication management was concerns regarding the medical understanding of mental health problems, which the service users related to feelings of lacking a voice, being spoken down to and being part of a culture where the doctor knows best [11]. The same study found that both service users and psychiatrists saw the legal context and fear of coercion as a barrier to SDM as this could hinder honest dialogue and the establishment of trust. Further barriers among patients may be a lack of interest in decision-making due to negative symptoms [21] or experiences of powerlessness that even many years later prevent them from expressing their ideas and preferences [22].

Patients receiving mental health care in Norway have the right, as far as is reasonable, to choose between different mental health treatment options, including treatment without medication [23], such as family interventions or cognitive behavioural therapy [24, 25]. Despite this, recent studies show that SDM for patients with psychotic disorders is often limited to making choices between different types of antipsychotic medication [26, 27]. This highlights the importance of examining the association between SDM and patients’ perceptions of being respected for their desire not to use medication.

Research that explores potential associations between sociodemographic factors, patient and clinician-reported factors and the degree of SDM is valuable. Knowledge from such research can help to implement SDM by focusing the attention on factors that are important for mental health care to improve SDM. However, knowledge of factors associated with the use of SDM is inadequate [28].

A cross-sectional study of 846 psychiatric outpatients in Spain found that patients feel less involved in decision making with increasing age and that a positive attitude towards psychiatric medication is associated with a high degree of SDM [18].

A cross-sectional study of 992 patients in Norwegian mental health care found that those in involuntary treatment reported a significantly lower degree of SDM than those treated voluntarily, and that male patients reported a significantly lower degree of SDM than females [19]. They also found that patients with longer treatment duration (more than the median of 2.2 years) and patients that used medications for their mental illness reported a significantly lower degree of SDM than those with shorter treatment duration and those who did not use medications.

A European multicenter study of 213 health professionals and 588 patients with severe mental illness found that health professionals more frequently adopted SDM when patients with a psychotic disorder were younger, had less severe symptoms and better quality of life [29]. A mixed methods study of 78 participants with schizophrenia spectrum disorder in Australia found that higher personal wellbeing and higher treatment satisfaction were significantly associated with a higher degree of SDM [30].

While the studies mentioned above have found associations between patient characteristics and SDM, other studies in the mental health setting have found no associations between age, gender, diagnosis [31–33] race [31, 33], previous psychiatric contact [32] and SDM. Moreover, a recent review examined the association between patient-related characteristics and the observed and/or experienced occurrence of SDM about treatment in routine care [28]. The authors found that the association between many of the patient-related characteristics and SDM remains unclear, and suggested further research to determine which patient-related characteristics might be associated with SDM.

The challenges of implementing SDM also require further research [1]. This is especially pertinent for patients with a psychotic disorder, as they tend to report a lower degree of SDM than other patients in mental health care [18, 19]. Knowledge of the factors that may be associated with involvement in SDM for patients with psychotic disorders can enhance understanding and help to empower these patients in the decision-making process.

Aims.

The current study aims to investigate the reported degree of SDM among patients with a psychotic disorder in mental health specialist services and factors associated with the SDM score. This may narrow down some of the factors associated with SDM and mitigate the uncertainty. We therefore pose the following research questions:

1. To what extent do patients with a psychotic disorder in mental health specialist services report SDM?
2. Which sociodemographic and patient- and clinician-reported factors are associated with the reported SDM score?

## Methods

### Design

The study design is cross-sectional, using baseline data from a Norwegian cluster-randomized study on implementation of evidence-based practices for psychosis (Clinical trial: NCT03271242) [34].

### Setting

The current study recruited patients from 26 community mental health centers and 13 hospital departments in six of the 19 Norwegian health trusts. These six health trusts provided services to 38% of the population in urban and rural areas throughout the country. The 39 sites had patients with a psychotic disorder as a major target group and included acute psychiatric units, units for the treatment of psychotic disorders, and various outpatient and mobile units. The sites had multidisciplinary clinical staff.

### Participants

We included 305 patients from June 2016 to March 2017 from the 39 clinical sites. Data on the number of patients invited who declined to participate were not available. The reason for not collecting this data was mainly due to resource constraints, as we wanted to ease the load on the already busy health professionals who conducted recruitment and data collection. Participants were eligible for inclusion if they were 16 years (the legal age in Norway to consent to health care and participation in research) or older, under assessment or treatment for a psychotic disorder (ICD-10 F20-29) and if their clinician assessed that they had the capacity to make the decision to participate. The local coordinators at each participating site drew up a list of all relevant participants in treatment when the inclusion period started. Inclusion of these was spread over time while newly referred patients were included continuously. Health professionals who knew the participants provided oral and written information about the study before the invitation to participate.

### Data collection

Both participants and health professionals completed a questionnaire when the participants were included in the study. They were asked to fill out the questionnaire on the day of inclusion or a few days later. Participants were given a safe and quiet place to complete the questionnaire. Outpatients could take the questionnaire home and send it back to the clinic in a prepaid, sealed envelope when they had finished. The health professional responsible for the treatment also completed the questionnaire, but could do so in collaboration with other health professionals in the treatment team. Information on the profession or education of the individual health professionals who filled out the questionnaire was not collected, as this was not planned for use in the data analyses and that we encouraged that the assessments of the patients was informed by the total information that the team members had about the patient.

### Instruments and variables

#### Clinician-rated measures

Health professionals reported age, gender, education and ethnicity. They also recorded total time of patients’ contact with services, whether patients were under community treatment orders (CTOs), and whether they used medication. Finally, health professionals completed the Clinical Global Impression-Severity (CGI-S), a one-item measure to assess the severity of the patients’ mental illness within the last week on a scale from 1 (normal, not at all ill) to 7 (extremely ill) [35].

#### Patient-rated measures

The participants rated the question “How satisfied are you with your life overall?” on a scale from 1 to 7, which is item 1 (Life as a whole) in the Manchester Short Assessment of Quality of Life [36]. The participants also rated on a 5-point Likert scale the degree to which they had been respected for their desire not to use medication during the last six months. Global satisfaction with services was rated using the Client Satisfaction Questionnaire-8 (CSQ-8), which is a brief 8-item self-report questionnaire [37].

CollaboRATE is a 3-item validated measure of SDM [38, 39], shown to be useful across different clinical settings and patient populations [40]. The participants rated the following three items, which relate to information, preference and integration, on a scale from 0 (no effort was made) to 9 (every effort was made): (1) How much effort was made to help you understand your health issues? (2) How much effort was made to listen to the things that matter most to you about your health issues?, and (3) How much effort was made to include what matters most to you in choosing what to do next? [38]. The participants rated the items based on their contact with health professionals in the past six months or based on all their contact with health professionals if they had been in contact with the services for fewer than six months in total.

The collaboRATE manual describes mean score and top score as two approaches to scoring [41]. The mean of the three responses for each participant provides a score between 0 and 9, where a higher score indicates more SDM. When calculating the top score, all participants with the highest response (i.e., 9) on all three items were given code 1 and all other participants were given code 0. The collaboRATE top score was the percentage of all participants coded as 1.

The top score represents the proportion of participants who felt that they received the highest possible quality SDM or a gold standard SDM compared to those who felt there was room for improvement [41, 42]. Previous studies show that also a sum score and a percentage score of the sum score are used [19]. The sum score is the sum of the three questions and provides a score between 0 and 27 where higher scores represent more SDM. The sum score percentage is the sum score converted to a percentage score by multiplying the sum score with 100/27 resulting in a range of 0-100. Both of these methods are a linear combination of each other and of the mean score. We decided to present descriptive data using all four scoring methods to facilitate comparison with other studies. The mean score was used in the regression analysis to retain information and statistical power.

#### Use of variables in the analysis

Age was used as a continuous variable in the analysis. Gender was coded as male or female and ethnicity dichotomized as Norwegian or other. Education was dichotomized as no/lower education (up to high school or equivalent) or higher education (1–6 years at college or university). Total time in contact with services was recorded as less than 6 months, 6–23 months, 2–5 years, 6–10 years or more than 10 years. CTO was recorded as yes or no. CGI-S was dichotomized into normal/borderline/mildly ill/moderately ill and markedly ill/severely ill/extremely ill. Whether the participant used medication was recorded as yes or no. We categorized quality of life as very much dissatisfied/much dissatisfied/quite dissatisfied, both dissatisfied and satisfied or quite satisfied/much satisfied/very much satisfied. Responses to the question on the desire not to use medication were categorized as strongly agree, agree, both agree and disagree, disagree or strongly disagree. CSQ-8 was used as a continuous variable ranging from 8 to 32 with higher scores representing more satisfaction with services.

### Statistical analysis

Frequencies and percentages for categorical variables and means and standard deviations (SDs) for continuous variables were used to describe the sample and extent to which the participants reported that SDM took place.

Imputation of missing values in CollaboRATE was performed on cases with fewer than 50% missing values on its items, i.e., CollaboRATE cases with one item missing were imputed. The empirical distribution for each item in the scale was generated. A random number was drawn from that distribution and used to replace the missing value. The process was repeated until all missing values were imputed. Three cases with one missing value, constituting < 1%, were imputed.

Bivariate and multiple linear mixed models were estimated to assess characteristics associated with SDM score. Random effects for units were included into the model as there was a clear cluster effect at unit level with an intra-class correlation coefficient of 0.13. No cluster effect of importance was found at health trust level, and this was therefore not adjusted for. The linear mixed models were estimated for participants with no missing values on the included independent variables (n = 233). Model assumptions were assessed by standard methods, and no major deviations were identified. Results were presented as regression coefficients with corresponding 95% confidence intervals and p-values. The participants with complete data on all assessed covariates were compared with those excluded from the regression analysis due to missing values based on an independent samples t-test or χ^2^-test.

Results with p-values < 0.05 were considered statistically significant and all tests were two-sided.

Analyses were conducted in IBM SPSS Statistics for Windows, version 26 (IBM Corp., Armonk, NY, USA) and STATA v 16.

### Ethical considerations

All participants voluntarily provided written informed consent. The study was approved by the Regional Committee for Medical and Health Research Ethics (REC South-East, Reg. No. 2015/2169) and by the data protection officer of each participating health trust. The study followed the principles of the Declaration of Helsinki.

## Results

### Patient characteristics at inclusion

The sociodemographic and clinical characteristics of the participants are shown in Table 1.

**Table 1:** Sociodemographic and clinical characteristics of the participants (n = 305)

Characteristics	
Age, mean (SD)	40 (12.6)
Gender, n (%)	
Female	126 (41)
Ethnicity, n (%)	
Norwegian	264 (88)
Other	36 (12)
Education, n (%)	
Lower education	226 (79)
Higher education	59 (21)
The Clinical Global Impression Severity, n (%)	
Normal/borderline/mildly/moderately	175 (61)
Markedly/severely/extremely	114 (39)
Total time in contact with services, n (%)	
Less than 6 months altogether	19 (6.5)
6–23 months	27 (9.2)
2–5 years	48 (16.4)
6–10 years	61 (20.8)
More than 10 years	138 (47.1)
Community treatment orders, n (%)	
Yes	41 (14)
No	258 (86)
Whether patient uses medication, n (%)	
Yes	281 (94.6)
No	16 (5.4)
Quality of life, n (%)	
Very much/much/quite dissatisfied	54 (17.7)
Both satisfied and dissatisfied	99 (32.5)
Quite/much/very much satisfied	152 (49.8)
Respected for desire not to use medication, n (%)	
Strongly disagree	63 (22)
Disagree	62 (22)
Both agree and disagree	60 (21)
Agree	56 (20)
Strongly agree	43 (15)
The Client Satisfaction Questionnaire-8, total score, mean (SD)	25.4 (4.8)

The participants were on average 40 (SD 12.6) years old, 41% were female, 88% were of Norwegian ethnicity and 79% had lower education. As for the severity of their illness, 39% were markedly/severely/extremely ill. Almost half of the participants had more than 10 years total time in contact with services and 14% were under a CTO. Medication was used by 95% of the participants, half of the participants were satisfied with their quality of life, 34% agreed that they were respected for their desire not to use medication and the CSQ-8 total score was 25.4 (SD 4.8).

### Patient-reported SDM

Table 2 presents descriptive statistics for collaboRATE mean score, top score, sum score, sum score percentage and item scores.

**Table 2:** CollaboRATE scores: Descriptive statistics for total scores and for items (N = 305)

	Mean (SD)	N (%)
**Collaborate total scale**		
Collaborate total mean score	6.3 (2.2)	
Collaborate top score		43 (14.1)
Collaborate sum score	18.8 (6.5)	
CollaboRATE sum score recalculated to a scale 1-100	69.8 (23.9)	
**CollaboRATE items**		
1. How much effort was made to help you understand your health issues?	6.4 (2.3)	
2. How much effort was made to listen to the things that matter most to you about your health issues?	6.4 (2.3)	
3. How much effort was made to include what matters most to you in choosing what to do next?	6.1 (2.4)	

The CollaboRATE mean score was 6.3 (max. 9), 14.1% scored nine on all three items (top score), the CollaboRATE sum score was 18.8 and the sum score percentage was 69.8. Item 1, which relates to information, had a mean score of 6.4, item 2 relates to preference and was scored at 6.4 and item 3, which relates to integration, had a mean score of 6.1.

### Factors associated with reported SDM score

Table 3 shows the results of the linear mixed model assessing the association between SDM and the patient- and clinician-rated measures.

**Table 3:** Results of linear mixed model for the association between covariates and SDM (n = 233)

Variable	Bivariate models			Multiple model		
	RC	95% CI	p	RC	95% CI	p
Age Gender (Male – ref.) Education (Lower – ref.) Ethnicity (Norwegian – ref.)	-0.01 0.32 0.25 1.03	(-0.04; 0.01) (-0.22; 0.86) (-0.39; 0.89) (0.18; 1.88)	0.234 0.243 0.449 **0.017**	-0.004 0.0001 0.23 0.63	(-0.02; 0.02) (-0.43; 0.43) (-0.28; 0.74) (-0.04; 1.30)	0.694 1.000 0.383 0.067
Total time in contact with services						
Less than 6 months altogether 6–23 months 2–5 years 6–10 years	0.52 -0.09 -0.13 0.18	(-0.69; 1.74) (-1.16; 0.99) (-0.89; 0.63) (-0.53; 0.89)	0.397 0.873 0.731 0.616	0.27 0.25 -0.02 0.11	(-0.67; 1.21) (-0.61; 1.11) (-0.65; 0.62) (-0.47; 0.69)	0.576 0.567 0.957 0.708
More than 10 years – ref.	0			0		
Community treatment order (No – ref.)	-0.88	(-1.67; -0.10)	**0.027**	-0.11	(-0.73; 0.52)	0.739
CGI-S						
Normal/borderline/mildly/moderately – ref.	0			0		
Markedly/severely/extremely Whether patient uses medication (Yes – ref.)	-0.53 -0.05	(-1.08; 0.03) (-1.29; 1.20)	0.062 0.942	-0.39 -0.02	(-0.83; 0.06) (-1.02; 0.98)	0.092 0.969
Quality of life						
Extremely/very/mostly dissatisfied Both satisfied and dissatisfied	-0.79 -0.54	(-1.53; -0.04) (-1.13; 0.04)	**0.038** 0.069	-0.20 -0.11	(-0.80; 0.39) (-0.57; 0.36)	0.502 0.651
Extremely/very/mostly satisfied – ref.	0			0		
Respect for desire not to use medication						
Strongly disagree – ref.	0			0		
Disagree Both agree and disagree Agree Strongly agree Total score CSQ-8	0.11 0.79 0.97 1.48 0.29	(-0.67; 0.90) (-0.01; 1.59) (0.16; 1.78) (0.63; 2.33) (0.25; 0.33)	0.774 0.054 **0.019** **0.001** **< 0.001**	0.10 0.53 0.25 0.50 0.27	(-0.52; 0.73) (-0.09; 1.16) (-0.39; 0.89) (-0.18; 1.19) (0.23; 0.32)	0.745 0.095 0.441 0.150 **< 0.001**

RC – regression coefficient; CI – confidence interval

According to the bivariate analyses, those with other ethnicity as compared to Norwegian ethnicity and those who were not under a CTO regime as compared to those on CTO regime had a statistically significantly higher SDM score. Further, those who were satisfied with their life compared to those who were dissatisfied and those who agreed or strongly agreed that they had been respected for their desire to not use medication as compared to those who strongly disagreed had a significantly higher SDM score. Finally, greater global satisfaction with services based on the CSQ-8 total score was associated with a higher SDM score.

No significant differences were found between participants included in the mixed models and those who were not included.

In the multiple linear mixed model, greater satisfaction with services was significantly associated with a higher SDM score. None of the other independent variables was associated with SDM in the multiple model.

## Discussion

The current study found that patients with psychotic disorders reported an SDM mean score of 6.3 and that 14.1% scored the highest response on all three questions in CollaboRATE (top score). The study also showed that higher global satisfaction with services was associated with higher SDM score.

The top score of 14.1% suggests that, despite a mean score of 6.3, few patients felt that a SDM gold standard was being practiced, namely a practice where every effort was made to include them in the decision-making process. This indicates that many patients found room for improvement.

The results in the current study on the extent to which patients reported that SDM had occurred are similar to results from an Australian study [30] which found that patients with schizophrenia (n = 78) reported a CollaboRATE sum score of 15.3 (18.8 in our study). Our results are also in line with a Norwegian study [19] where the participants with a psychotic disorder (82 out of a sample of 992) had a CollaboRATE percentage sum score of 66.8 and a top score of 11% (69.8 and 14.1%, respectively, in our study).

The whole sample in that Norwegian study [19] consisted of 992 people with different mental health diagnoses and reported a CollaboRATE percentage sum score of 80.7 and a top score of 27.4%. A study from the Canary Islands included 191 psychiatric outpatients with different diagnoses and found a CollaboRATE sum score of 22.6 and a top score of 39.8 [43]. Comparing these results with findings from the current study reveals that the patients with psychotic disorders in our study reported a considerably lower degree of SDM than has been reported from groups of people with various mental health diagnoses. This finding is supported by previous studies showing that patients with a psychotic disorder perceived lower degrees of SDM than patients with other mental health diagnoses [18, 19]. The difference in reported SDM is even larger when our results are compared to studies in primary care clinics where CollaboRATE top scores of between 61% and 86% have been reported [40, 44].

The current study found CollaboRATE item scores of 6.4 (Item 1), 6.4 (Item 2) and 6.1 (Item 3). Item 3 had a slightly lower score than the first two items, suggesting that health professionals may be a little better at giving information and at eliciting preferences than at integrating patient preferences. The previously mentioned study from the Canary Islands [43] found item mean scores of 7.5 (Item 1), 7.6 (Item 2) and 7.4 (Item 3), which shows that the patients in the current study perceived slightly lower levels of information, preference elicitation and integration. This suggests that patients with psychotic disorders also experience a lower degree of SDM than do groups consisting of people with different mental health diagnoses, when sub-elements of SDM are examined.

A possible explanation for the difference in SDM between patients with psychotic disorders and other patients in mental health care could be that it is more challenging to practice SDM for those with serious mental illness who often require more comprehensive and long-term treatment. However, if this was the case, it would be reasonable to assume that the severity of the patients’ illness would also be associated with SDM, which was not found in the current study.

It may also be that the different treatment settings (community mental health centers, hospital departments, inpatient and outpatient settings) cause some of the difference in scores, e.g., outpatients experience more SDM than patients at other levels of care, which is also shown in previous research [19]. The current study included both inpatients and outpatients, but does not have data on how many of the patients received treatment at each level of care.

Another explanation could be the patients’ decisional capacity, as previous research has shown that health professionals consider lack of decisional capacity as a major barrier to SDM for patients with psychotic disorders [20]. We acknowledge that SDM, during certain periods, can be more challenging for patients with psychotic disorders than for those with less severe illnesses, due to various factors, such as the severity of symptoms such as hallucinations and delusions, cognitive impairments, patients’ level of insight into their condition, and their ability to effectively communicate and participate in decision-making. Acute psychosis is an example of a situation where the patient may not necessarily know what the best choice is, which requires the health professional to make a decision that may not align with the patient’s preference.

However, previous studies also show that a considerable number of patients with psychotic disorders have adequate decision-making capacity [45–47]. This suggests that it may be possible to include these patients in decisions about their own health care to a greater extent than in current practice, and that part of the reason for lower SDM among patients with psychotic disorders may be that health professionals are hesitant to involve them.

The difference is also interesting in light of previous research findings showing that having a schizophrenia diagnosis and more serious symptoms was associated with a greater probability of coercion [48]. If patients with psychotic disorders receive coercive treatment to a greater extent than other patients in mental health care, this may help to explain their lower levels of SDM, which is also supported by a previous study showing that involuntary treatment was associated with lower SDM [19].Fourteen percent of the patients in the current study were under a CTO. The bivariate analysis showed that those who were not subject to a CTO had a significantly higher SDM score than those who were, but this association was not significant in the analyses that adjusted for the other covariates. The current study did not include data on admission status or involuntary treatment. The association between these factors and SDM should therefore be further investigated in future studies.

The multiple linear mixed model showed that global satisfaction with services was significantly positively associated with SDM. This is in line with a previous survey of patients with schizophrenia spectrum disorder [30]. It is also in accordance with a randomized controlled trial of two community-based treatment programs for patients with schizophrenia, where it was found that the program that included procedures for SDM showed significantly greater satisfaction than the program consisting of the established mode of treatment [49]. Further, a qualitative study of mental health pharmacists’ views on SDM for antipsychotics in serious mental illness revealed similar findings, as the pharmacists felt that SDM could increase service user satisfaction [50]. The finding is also supported by a review of 39 studies from a variety of clinical contexts showing that patients who report having participated in SDM are likely to have greater satisfaction [12].

A possible explanation for this finding may be the potential benefits of SDM for patients. For example, when patients are involved in making health care choices, they may feel more in control of their care and more confident in the treatment choices they make. This can lead to greater satisfaction with the treatment, as they find that their values and preferences are respected. This is interesting in relation to a previous study, which found that when patients were more involved in the decision-making process than they preferred to be, they were more satisfied [51]. This emphasizes the need to define roles and the patients’ desire for involvement as part of the SDM process [8]. When the patients’ preference for involvement is clarified, health professionals can facilitate their care in accordance with the patients’ specific needs and this may lead to more personalized care and greater satisfaction.

Another possible explanation may be that patients experience better health outcomes when SDM is practiced and are therefore more satisfied with their treatment. As the current study did not include any measure of clinical health outcomes, we were unable to draw any conclusion on a potential association between SDM and health outcomes. Furthermore, these factors may be linked in a different way. For example, improved patient satisfaction may be a result of SDM, which in time may lead to trust in the health professional, accompanied by adherence to the health professional’s recommendations and finally improved health. However, more knowledge is needed to come to a conclusion about the connection between these factors [12].

It is interesting that the results in the current study are not in line with earlier findings, which suggests that patients with longer treatment duration report lower SDM than those with shorter treatment duration [19]. A review found that a possible barrier to SDM in prescribing antipsychotics is that patients did not want an active role in their medication and instead took a passive approach, partly because of past experience of being denied involvement [52]. In line with this, a possible expectation in the current study could have been that patients’ experience of clinician-led mental health care may have created a self-image of a passive patient with little to contribute in decisions. However, longer time in contact with services may also help to build trusting relationships, which are said to be an important part of SDM in mental health [53]. The association between time in contact with services and SDM should be investigated in future studies.

The current study aligns with several previous studies conducted in different contexts showing that satisfaction is associated with SDM. This association highlights the potential role of SDM in providing person-centered care that lead to patient satisfaction.

### Limitations

The current study has several potential limitations. First, we did not randomly select the participants. Their experiences may thus not accurately represent the population of patients with psychotic disorders in Norway. Second, although a list was drawn up of all relevant patients eligible for participation, we do not have information on how many patients were actually invited. Thus, it may have been a convenience sample, which limits the generalizability of the findings. Third, asking participants to complete CollaboRATE based on their contact with health professionals in the last six months may have led to recall bias. Fourth, a longer measure of the SDM process could have provided more details. However, as this study was part of a larger multicenter study, CollaboRATE was considered suitable because it is a brief measure with three key dimensions of SDM [38] that is quick to complete [39, 43]. Finally, as this was a cross-sectional study, we were not able to draw any conclusions regarding causality.

## Conclusions

The current study shows that patients with psychotic disorders reported lower levels of SDM than in studies of groups consisting of people with various mental health diagnoses. This indicates a potential to design services that enable patients with psychotic disorders to play a larger role in decision-making, although it is not given that this group will always be able to participate in decision-making at the same level as those with less severe illnesses. The positive association between global satisfaction with services and SDM highlights the potentially vital role of SDM in providing person-centered care which leads to patient satisfaction.

## Data Availability

The dataset used and/or analysed during the current study is available from the corresponding author on reasonable request.

## Abbreviations

SDM: Shared decision-making
CTO: Community treatment orders
CGI-S: The Clinical Global Impression Severity
CSQ-8: The Client Satisfaction Questionnaire-8
